# Effects of RAAS blockade on acute kidney injury in head and neck cancer patients post-chemoradiotherapy

**DOI:** 10.1186/s12882-025-04195-z

**Published:** 2025-06-03

**Authors:** Yi-Ting Chen, Yao-Hung Kuo, Chih-Feng Lin, Chun-Wei Wang, Chiao-Ling Tsai

**Affiliations:** 1 Division of Nephrology, Department of Internal Medicine, National Taiwan University Hospital, Taipei, Taiwan; 2 Division of Blood Purification, Department of Integrated Diagnostics & Therapeutics, National Taiwan University Hospital, Taipei, Taiwan; 3 Department of Radiation Oncology, E-Da Hospital, I-Shou University, Kaohsiung, Taiwan; 4 Department of Otolaryngology, National Taiwan University Hospital, Taipei, Taiwan; 5 Division of Radiation Oncology, Department of Oncology, National Taiwan University Hospital, Taipei, Taiwan; 6 Department and Graduate Institute of Medical Education and Bioethics, National Taiwan University College of Medicine, No. 1, Sec. 1, Ren’ai Rd., Zhongzheng Dist., 100, Taipei, Taiwan

**Keywords:** Acute kidney injury, RAAS blockade, Head and neck cancer

## Abstract

**Background:**

The incidence of acute kidney injury (AKI) is high among head and neck cancer (HNC) patients following Platinum-based concurrent chemo-radiotherapy (CCRT). However, the effect of renin-angiotensin-aldosterone system (RAAS) blockade on the risk of AKI in HNC patients undergoing CCRT is controversial. This study aimed to investigate the association between RAAS blockade, AKI and survival in HNC patients undergoing CCRT.

**Method:**

This retrospective cohort study included 989 HNC patients treated between January 2016 and July 2022, with follow-up extending to July 2022. Among them, 65 (6.6%) patients were using RAAS blockade for hypertension control, while 924 were non-users. Clinical data and demographics were retrieved. Cox regression models were employed to analyze primary outcomes, including AKI and patient survival.

**Results:**

There were 65 (6.6%) patients being RAAS blockade users in the study. The mean age of RAAS blockade users was older than that of non-users (61 vs. 55 years old, p < 0.001). Overall, 219 (22.1%) patients developed AKI, including 25 RAAS blockade users. RAAS blockade users had a higher risk of AKI compared to non-users (38% vs. 21%, p = 0.001) and also had a worse mortality rate (35% vs. 22%, p = 0.015). Factors such as male gender, age, RAAS blockade usage, and baseline serum creatinine levels independently predicted the onset of AKI and patient survival.

**Conclusion:**

RAAS blockade users developed AKI, which significantly predicted patient survival. Diligent post-CCRT renal function monitoring and hydration in RAAS blockade users are crucial to mitigate AKI risk and potentially improve survival in this patient group.

## Introduction

Platinum-based chemoradiotherapy (CCRT) targeting the oropharynx often induces mucositis, dehydration, and malnutrition in head and neck cancer (HNC) patients, increasing the risk of acute kidney injury (AKI) [1, 2]. Approximately 60–75% of HNC patients receiving radiotherapy (RT) develop swallowing dysfunction [3,4]. RT doses exceeding 50Gray significantly increase the risks of aspiration and stricture formation, further aggravating volume depletion [5].

Previous studies reported that cisplatin-based CCRT increases the incidence of AKI by 4–8% [6, 7]. One retrospective study found that 69% of patients with advanced HNC undergoing high-dose cisplatin CCRT developed AKI, leading to significant long-term renal impairment [8]. Comorbidities such as hypertension, congestive heart failure, and diabetes also increase the potential risk of AKI [8].

RT-induced kidney damage includes vascular, glomerular and tubulointerstitial damage. The mechanisms of kidney injury due to RT include:

1. Direct nephrotoxicity, where ionizing radiation causes double-stranded DNA breaks, leading to cell death, including apoptosis and necrosis of renal endothelial, tubular and glomerular cells.
2. Inflammation, involving oxidative stress, cellular senescence, activation of the renin-angiotensin-aldosterone-system (RAAS).
3. Vascular damage [9, 10].

RAAS blockade, including angiotensin-converting enzyme inhibitor (ACEi) and angiotensin receptor blocker (ARB), is used to treat hypertension, decrease proteinuria, organ prevention such as cardiovascular disease and progression of chronic kidney disease [11, 12]. The activation of the intrarenal RAAS in patients with AKI is associated with the severity of the condition and plays a critical role in hemodynamic regulation and the progression of kidney disease [13, 14]. RAAS blockade may exacerbate AKI, especially in patients with hypovolemia, renal artery stenosis or low eGFR and may lead to increased hospital admissions due to AKI [13, 15]. However, RAAS blockade can also suppress the production of reactive oxygen species, enhance the generation and bioactivity of nitric oxide, improve renal cortical microvascular oxygenation, and help restore endothelial function [16, 17].

Previous reports indicate that RAAS blockade offers protective benefits against contrast-associated AKI, especially in advanced chronic kidney disease [18]. Conversely, one retrospective cohort study reported that ACEi use, alone or combined with body weight loss over 10%, caused increased renal toxicity in patients with stage III–IVB HNC during CCRT, without affecting their disease control or survival [19]. Similarly, another study found that patients using ACEi were predisposed to AKI when combined with volume depletion, such as from diuretics [20]. Thus, the effect of RAAS blockade remains controversial.

This study aims to investigate the effect of RAAS blockade on the risk of AKI and mortality in HNC patients undergoing CCRT.

## Methods

### Study design and population

This is a retrospective hospital-based cohort study at the National Taiwan University Hospital (NTUH), a tertiary medical center in Taiwan. We reviewed patients older than 20 years old with HNC who received CCRT between January 2016 and July 2022 at NTUH. The study was reviewed and approved by the Institutional Review Board of National Taiwan University Hospital (IRB No. 202210026RINC), and its protocol adheres to the Declaration of Helsinki.

The data of the study population were extracted from the integrated medical Database (NTUH-IMD). The NTUH-iMD contains de-identified electronic medical records from NTUH. This allowed us to access the medical records of these individuals from 2016 to 2022, including demographic information (age, gender, and body mass index (BMI)), comorbidities (diabetes, stroke, and cardiovascular disease), baseline renal function, and renal function change during CCRT, dialysis, tumor stage, chemotherapy regimen, and death. The clinical outcome data were meticulously collected and monitor until July 2022, with the primary outcomes focusing on acute kidney injury and mortality during and after CCRT. The diagnostic criteria for HNC encompass malignancies originating in several anatomical subsites, including the oral cavity (comprising the buccal mucosa, gingiva, floor of mouth, and palate), oropharynx (including the tonsils), nasopharynx, hypopharynx, larynx (including the glottis), and the major and minor salivary glands.

### The CCRT protocols

The concurrent chemotherapy regimens included platinum administered weekly (cisplatin, 30–40 mg/m^2^) during radiotherapy. Chemotherapy was withheld until nadir values were ≥ 1500/μL for neutrophils and ≥ 100,000/μL for platelets. All patients were irradiated with volumetric-modulated arc therapy (VMAT). Radiotherapy was given as 2.0–2.12 Gy per fraction with five daily fractions per week for 6–7 weeks, for a total dose of 60–70 Gy to the primary tumor ± high-risk region and 54–60 Gy to the involved neck area.

### Definitions of RAAS blockade user

RAAS blockade users were defined as patients who have been prescribed and had used

ACEi/ARBs for more than 90 days. The prescription date, dosage, and frequency of use were recorded.

### Definitions of acute kidney injury (AKI)

Serum creatinine (sCr) levels were obtained from the clinical laboratory database at baseline (the day before starting CCRT), weekly during CCRT, and six months after completing CCRT. AKI was identified based on the kidney disease improving global ooutcome (KDIGO) criteria (an increase in sCr ≥ 0.3 mg/dL over 48 hours or an increase in sCr by ≥ 1.5 folds from baseline occurring over 7 days) [21, 22].

### Statistical analyses

Data were presented as mean (with a 95% confidence interval), median (with the range), or number (as a percentage).

Logistic regression models were used analyzed renal function over time and to identify predictors for AKI.

The Kaplan-Meier curves were constructed for survival. Initial determination of outcome predictors was conducted through univariate analysis, followed by the inclusion of significant variates identified in the univariate analysis in the multivariate Cox regression analysis. The statistical analyses were performed using SAS software, version 9.4 (SAS Institute Inc). A significance level of P < 0.05 was applied. Results were presented as odds or hazard ratios with their corresponding 95% confidence intervals (CI).

## Results

### Patient characteristics

**Fig. 1:**
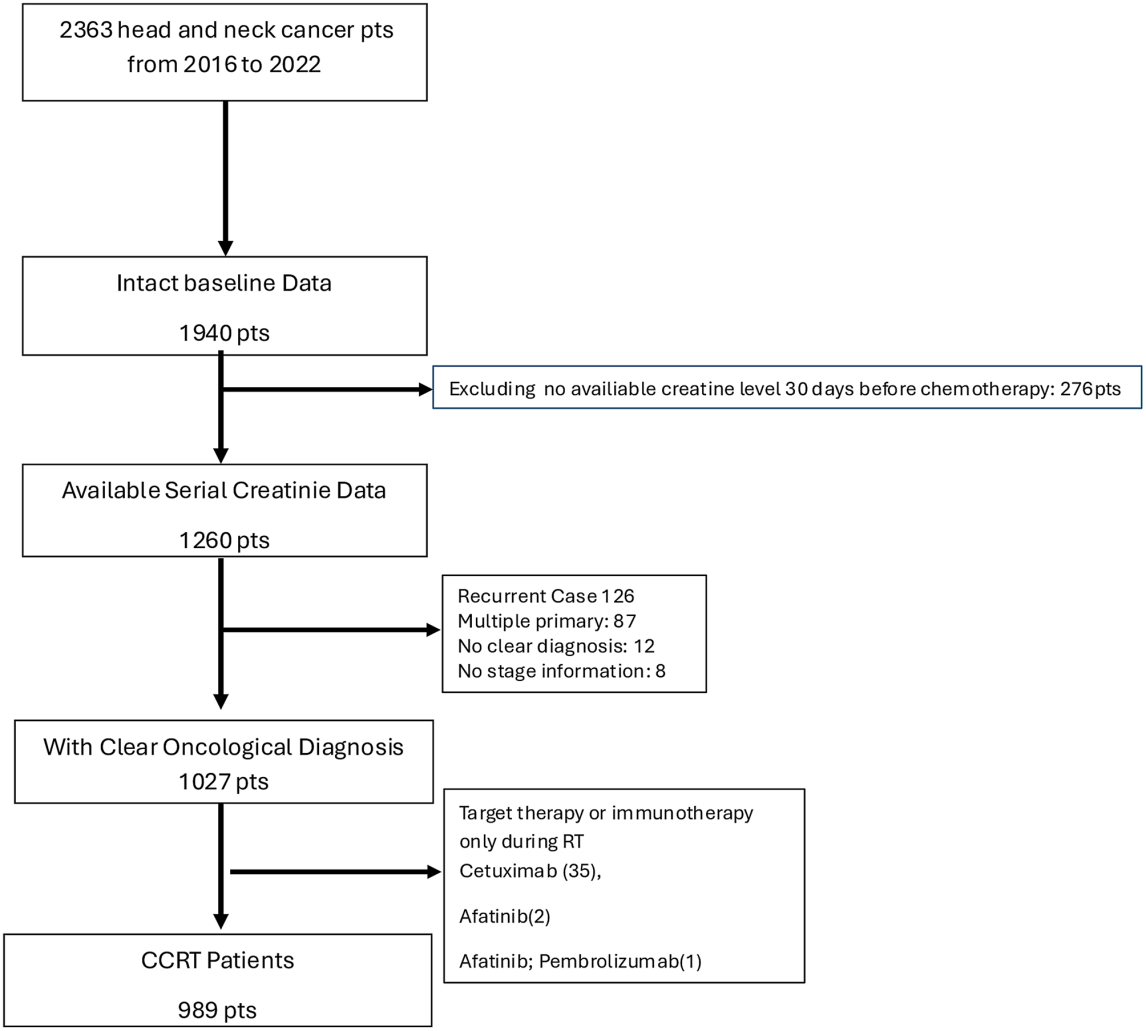
Flow diagram of all participants

Figure 1 showed flow diagram of this study. A total of 989 patients were included in the study, comprising 65 (6.6%) HNC patients who took RAAS blockade for hypertension control and 924 (93.4%) patients were non-users of RAAS blockade. Most of them were males. The median age of RAAS blockade users were 61 years (56, 58), which was older than non-users (median age 61 vs. 55 years old, *P* < 0.001).

Table 1 presents the baseline characteristics of the patients. Baseline serum creatinine levels were higher in RAAS blockade users compared to non-users (median level 0.90, inter-quartile range, IQR 0.80–1.10 vs. 0.90, 0.70–1.00, p = 0.017). RAAS blockade users also had a higher BMI than non-users (median 25.7 vs. 23.9 Kg/m2, p < 0.001). There was no statistical difference in Charlson index, tumor stage, RT dose, or chemotherapy regimen between RAAS blockade users and non-users. The median follow-up period was also not different between RAAS blockade users and non-users (25 vs. 35 months, p = 0.1).

**Table 1:** Baseline characteristics of all patients

Baseline characteristic	All participants, N = 989	**Non-users**, N = 92^1^	**RAAS blockade users**, N = 65^1^	**p-value**^2^
**Gender**				0.2
F	174 (18%)	166 (18%)	8 (12%)	
M	815 (82%)	758 (82%)	57 (88%)	
**Age**	56 (47, 63)	55 (47, 63)	61 (56, 68)	**<0.001**
**Baseline Creatinine (mg/dL)**	0.90 (0.70, 1.00)	0.90 (0.70, 1.00)	0.90 (0.80, 1.10)	**0.017**
**Charlson**	2.00 (2.00, 2.00)	2.00 (2.00, 2.00)	2.00 (2.00, 2.00)	0.14
Unknown	1	1	0	
**T stage**				0.3
0	3 (0.3%)	3 (0.3%)	0 (0%)	
1	158 (16%)	151 (16%)	7 (11%)	
2	231 (23%)	218 (24%)	13 (20%)	
3	198 (20%)	188 (20%)	10 (15%)	
4	399 (40%)	364 (39%)	35 (54%)	
**N stage**				0.055
0	255 (26%)	237 (26%)	18 (28%)	
1	208 (21%)	193 (21%)	15 (23%)	
2	341 (34%)	313 (34%)	28 (43%)	
3	185 (19%)	181 (20%)	4 (6.2%)	
**M stage**	5 (0.5%)	5 (0.5%)	0 (0%)	>0.9
**BMI**	24.0 (21.5, 26.6)	23.9 (21.3, 26.5)	25.7 (23.3, 29.0)	**<0.001**
**RT dose**	7,000 (6,600, 7,000)	7,000 (6,600, 7,000)	7,000 (6,600, 7,000)	0.7
**Chemotherapy Regimen**				0.2
Fluorouracil	19 (1.9%)	16 (1.7%)	3 (4.6%)	
Others	11 (1.1%)	11 (1.2%)	0 (0%)	
Platinum	911 (92%)	853 (92%)	58 (89%)	
Platinum_Doublet	27 (2.7%)	26 (2.8%)	1 (1.5%)	
Platinum_VEGF	21 (2.1%)	18 (1.9%)	3 (4.6%)	
**Cisplatin Monotherapy**				0.4
Others	78 (7.9%)	71 (7.7%)	7 (11%)	
Platinum	911 (92%)	853 (92%)	58 (89%)	
**Acute kidney Injury**	219 (22%)	194 (21%)	25 (38%)	**0.001**
**Death**	228 (23%)	205 (22%)	23 (35%)	**0.015**
**Follow-up period (Months)**	34 (15, 54)	35 (15, 55)	25 (13, 44)	0.10

^1^ n (%); Median (Q1, Q3)

^2^ Pearson’s Chi-squared test; Wilcoxon rank sum test; Fisher’s exact test Abbreviation: F, female; M, male; T, tumor; N, lymph node; M, metastasis; BMI, body mass index; RT, radiotherapy; VEGF, vascular endothelial growth factor

### Predictors of acute kidney injury post-CCRT

A total of 219 (22.1%) patients experienced acute kidney injury (AKI) events. The risk of AKI was significantly higher in RAAS blockade users compared to non-users (38% vs. 21%, p = 0.001) (Table 1). Univariate analysis of baseline characteristics showed that age, RAAS blockade use, baseline serum creatinine levels, and platinum based chemotherapy regimen were risk factors for AKI (p < 0.001, = 0.001, 0.016, and 0.03, respectively). The multivariate logistic regression model demonstrated that male gender, age, RAAS blockade use, and baseline serum creatinine levels were significantly associated with AKI during CCRT (p = 0.018, 0.009, 0.007, 0.01, and 0.019, respectively) (Table 2). RT dose was not significantly associated with AKI in univariate analysis (OR = 1.00, 95%CI; 1.00–1.00, p = 0.073), but emerged as a statistically significant predictor in the multivariate model (OR = 1.00, 95% CI; 1.00–1.00, p = 0.019).

**Table 2:** Predictors of acute kidney injury in univaraite / multivariate logistic regression

	Univariate				Multivariate		
Characteristic	N	OR^1^	95% CI^1^	p-value	OR^1^	95% CI^1^	p-value
**Gender**	989						
F		—	—		—	—	
M		0.75	0.52, 1.10	0.13	0.62	0.42, 0.93	**0.018**
**Age**	989	1.02	1.01, 1.04	**< 0.001**	1.02	1.00, 1.03	**0.009**
**RAAS blockade**	989	2.35	1.38, 3.95	**0.001**	2.11	1.21, 3.59	**0.007**
**Baseline Creatinine (mg/dL)**	989	1.52	1.10, 2.21	**0.016**	1.71	1.16, 2.61	**0.010**
**Charlson**	988	1.01	0.85, 1.17	> 0.9			
**T stage**	989	1.10	0.96, 1.26	0.2			
**N stage**	989	1.01	0.88, 1.16	0.9			
**M stage**	989	0.00		> 0.9			
**BMI**	989	1.01	0.97, 1.04	0.8			
**RT dose**	989	1.00	1.00, 1.00	0.073	1.00	1.00, 1.00	**0.019**
**Cisplatin Monotherapy**	989						
Others		—	—		—	—	
Platinum		0.57	0.35, 0.96	**0.030**	0.75	0.44, 1.30	0.3

^1^n (%); Median (Q1, Q3)

^2^Pearson's Chi-squared test; Wilcoxon rank sum test; Fisher's exact test. Abbreviation: F, female; M, male; T, tumor; N, lymph node; M, metastasis; BMI, body mass index; RT, radiotherapy; VEGF, vascular endothelial growth factor

### Predictors for patient survival

**Fig. 2:**
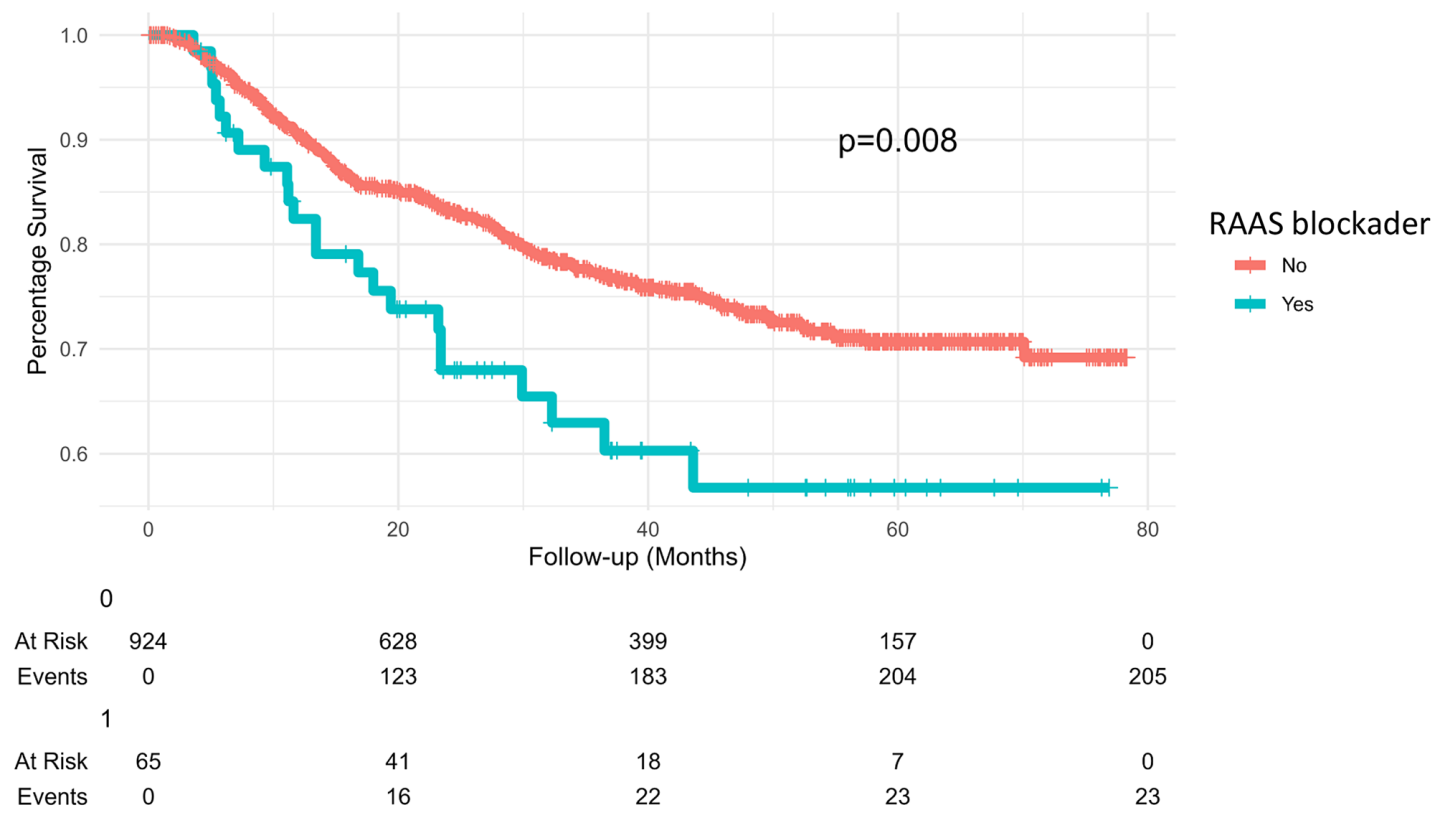
Kaplan-Meier plots of overall survive

During the follow-up period, 228 (23.1%) patients died. The medium follow-up period was not different between RAAS blockade users and non-users (25 vs. 35 months, p = 0.10). However, RAAS blockade users had a higher mortality rate compared to non-users (35% vs. 22%, p = 0.015) (Table 1). In univariate analysis of patient survival, male gender, patient age, RAAS blockade use, baseline serum creatinine, tumor size, lymph node metastasis, BMI, RT dose, platinum chemotherapy, and onset acute kidney injury were significant risk factors (p = 0.004, < 0.001, = 0.009, = 0.003, < 0.001, < 0.001, < 0.001, = 0.002, = 0.024, < 0.001, < 0.001, respectively). Multivariate cox regression analysis demonstrated that male gender, patient age, RAAS blockade use, tumor size, lymph node metastasis, BMI, RT dose, platinum chemotherapy, and onset of acute kidney injury were independent predictors for patient survival (p = 0.007, = 0.012, = 0.039, < 0.001, < 0.001, < 0.001, = 0.005, = 0.017, and < 0.001, respectively) (Table 3). The Kaplan-Meier survival analysis illustrated that RAAS blockade non-users had significantly better survival than RAAS blockade users (p = 0.008) (Fig. 2).

**Table 3:** Predictors of overall survival in univaraite / multivariate cox regression

	Univariate				Multivariate		
Characteristic	N	HR^1^	95% CI^1^	p-value	HR^1^	95% CI^1^	p-value
**Gender**	989						
F		—	—		—	—	
M		1.85	1.22, 2.80	**0.004**	1.78	1.17, 2.71	**0.007**
**Age**	989	1.03	1.01, 1.04	**< 0.001**	1.02	1.00, 1.03	**0.012**
**RAAS blockade**	989	1.78	1.15, 2.73	**0.009**	1.60	1.02, 2.51	**0.039**
**Baseline Creatinine (mg/dL)**	989	1.36	1.11, 1.66	**0.003**			
**Charlson**	988	0.99	0.85, 1.14	0.88			
**T stage**	989	1.40	1.23, 1.59	**< 0.001**	1.32	1.16, 1.51	**< 0.001**
**N stage**	989	1.30	1.15, 1.48	**< 0.001**	1.38	1.21, 1.57	**< 0.001**
**M stage**	989	0.82	0.11, 5.85	0.84			
**BMI**	989	0.95	0.92, 0.98	**0.001**	0.94	0.91, 0.98	**< 0.001**
**RT dose**	989	1.00	1.00, 1.00	**0.002**	1.00	1.00, 1.00	**0.005**
**Chemotherapy Regimen**	989						
Fluorouracil		—	—				
Others		1.11	0.33, 3.68	0.87			
Platinum		0.44	0.22, 0.90	**0.024**			
Platinum_Doublet		0.94	0.38, 2.34	0.90			
Platinum_VEGF		1.00	0.40, 2.54	> 0.99			
**Cisplatin Monotherapy**	989						
Others		—	—		—	—	
Platinum		0.45	0.31, 0.65	**< 0.001**	0.63	0.43, 0.92	**0.017**
**Acute kidney Injury**	989	1.76	1.33, 2.32	**< 0.001**	1.61	1.21, 2.14	**0.001**

^1^HR, Hazard Ratio; CI, Confidence Interval

Abbreviation: F, female; M, male; RAAS, Renin angiotensin system; T, tumor; N, lymph node; M, metastasis; BMI, body mass index; RT, radiotherapy; VEGF, vascular endothelial growth factor

## Discussion

Our study demonstrated that the risk of AKI in patients with HNC during CCRT was significantly associated with RAAS blockade use, advanced age, impaired baseline renal function (p < 0.05 for all factors). Interestingly, male sex exhibited a protective effect against AKI (OR = 0.62, 95% CI: 0.42–0.93, p = 0.018) but was identified as a risk factor for reduced overall survival (HR = 1.78, 95% CI: 1.17–2.71, p = 0.007).

The observed incidence rate of AKI in our cohort (22.1%) was comparable to the range reported in previous studies (31–43%) investigating similar patient populations undergoing CCRT for HNC [19, 23]. AKI occurred in 69% of patients undergoing high-dose cisplatin CCRT, partly due to the identification of low-stage AKI using the KDIGO criteria [8].

Cisplatin is a well-known nephrotoxin. Cisplatin will accumulate in proximal renal tubular epithelial cells, leading DNA damage, increase oxidative stress, mitochondrial damage, increase endoplasmic reticulum stress, autophagy activation, and cell-cycle dysregulation, resulting to acute kidney injury then chronic kidney disease [24]. Radiotherapy can worsen nephrotoxicity caused by cisplatin, particularly when the kidneys receive more than 2,300cGy of radiation in fractionated doses over 3–5 weeks, as it triggers oxidative stress and inflammatory processes within the kidneys [25].

Prior studies have reported risk factors for cisplatin-induced nephrotoxicity, including older age, hypertension, female gender, smoking, black ethnicity, hypokalemia, hypoalbuminemia, concomitant use of other anti-cancer medications, and single-dose versus fractionated dose RT [26–30]. The association of older age with AKI was confirmed in our study. However, Charlson index and tumor staging were not significant as a risk factor.

Baseline creatinine level was an independent predictor of AKI, a finding supported by other studies [8]. The univariate analysis showed no significant associated between RT dose and AKI (OR = 1.00, 95% CI: 1.00–1.00, p = 0.073). However, in the multivariate model, which accounts for potential confounding variates, RT dose emerged as a statistically significant predictor (OR = 1.00, 95% CI: 1.00–1.00, p = 0.019). The discrepancy between these results suggests that the relationship between RT dose and AKI may be influenced by other variables in the model. Patients receiving higher RT doses often have more complicated disease severities.

Unlike previous findings, male gender was a risk factor for AKI in our study, which may be due to the higher prevalence to smoking or/and alcohol consumption among male patients in Taiwan according to the National Health Administration’s 110^th^ National Health Interview Survey (the drinking rate in people aged 18 and over is 32% for men and 17.1% for women).

Few studies have discussed the benefit of RAAS blockade on the risk of AKI in HNC patients [19, 31]. In our study, we found that AKI was associated with RAAS blockade. The pathophysiology of AKI after CCRT is multifactorial. One possible cause is volume depletion. HNC patients often experience nausea and vomiting during CCRT. RAAS blockade can lower BP, increasing the risk of AKI if these patients do not receive adequate hydration and BP monitoring. The predominance of male patients (more than 80%) in our study is significant, as men are generally less attentive to physical changes such as BP, compared to female patients, especially those with a higher likelihood of smoking or/and alcohol consumption from the Taiwan National Health Administration.

The study has several limitations that warrant consideration. Firstly, its single-center, retrospective design may limit the generalizability of our findings. Secondly, the medical records lacked comprehensive documentation of potentially influential factors, including detailed weight fluctuations, hydration status, intravenous fluid administration, and nutritional intake patterns, which could have provided valuable context for our analyses. Thirdly, the relatively small sample size of 65 patients using RAAS blockade may have limited the statistical power of our analyses. However, these limitations are partially mitigated by the fact that our institution is a tertiary national medical center that receives referrals from other hospitals, positioning us as a major treatment center for HNC patients in Taiwan and across Asia. This status enhances the representativeness and reliability of our data within the regional context.

## Conclusion

In conclusion, AKI emerges as a frequent complication in patients with HNC following CCRT. Notably, the use of RAAS blockade warrants particular attention as it represents an independent risk factor contributing to both AKI incidence and mortality in this patient population. Our findings underscore the necessity for close monitoring of renal function during and post-CCRT to mitigate the risk of AKI, especially in patients with impaired baseline creatinine levels. Future research should focus on developing targeted preventive strategies, particularly for high-risk patients such as those using RAAS blockade medications.

## Data Availability

The datasets used and analyzed during the current study are available from the corresponding author on reasonable request.
